# Supplementary material for: The tumour suppressor CYLD regulates the p53 DNA damage response
Source: Nat Commun. 2016 Aug 26;7:12508. doi: 10.1038/ncomms12508 (PMC5007442; doi:10.1038/ncomms12508)
Supplement: Supplementary Information — Supplementary Figures 1-9 [file ncomms12508-s1.pdf]

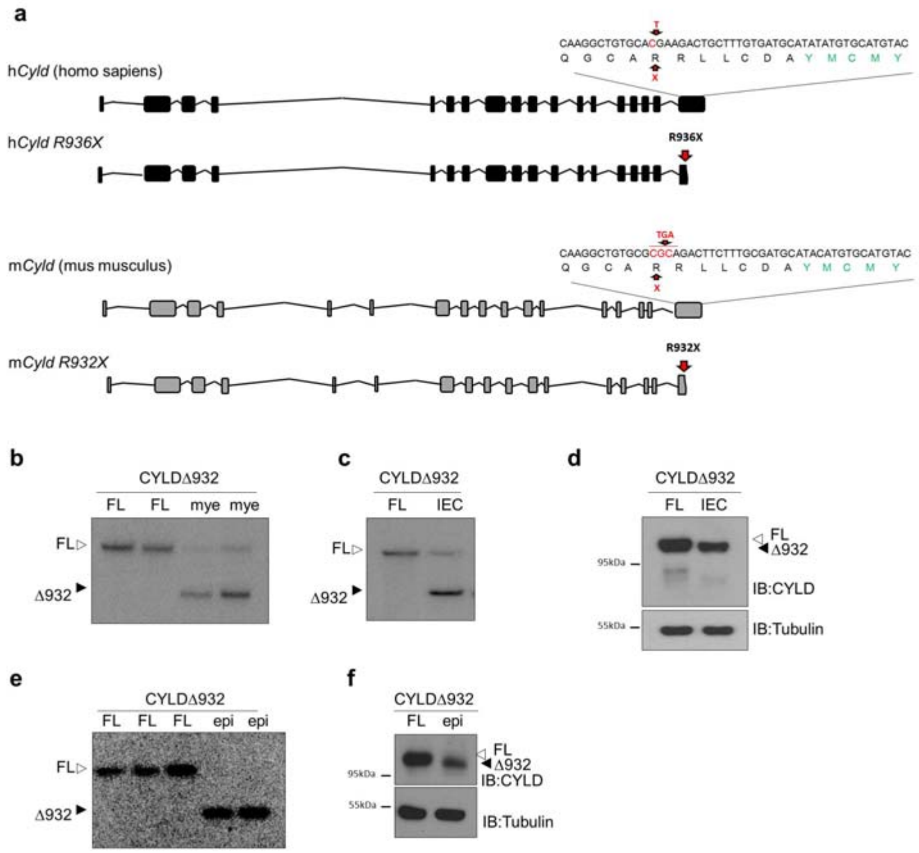

Supplementary Figure 1. Expression of catalytically deficient CYLD in intestinal epithelial cells, myeloid cells and epidermal keratinocytes

(a) Schematic representation of the human (h) and murine (m) wild type *Cyld* genes and the h*Cyld*R936X, m*Cyld*R932X mutants. The position and type of mutation are indicated in red. The subdomain III of the C-terminal histidine box sequence is shown in green. Analysis of genomic DNA isolated from bone morrow derived macrophages **(b)**, primary intestinal epithelial cells **(c)** and epidermal keratinocytes **(e)** prepared from CYLD$\Delta932^{FL}$ and CYLD$\Delta932^{mye}$, CYLD$\Delta932^{IEC}$ or CYLD$\Delta932^{epi}$ mice respectively show efficient deletion of the loxP-flanked genomic CYLD fragment. DNA was digested with SpeI and the probe used was amplified using the following primers, sense: 5’TCA TGG CCA GCA GTC TCG AAG3’; anti-sense: 5’TTT CTG TGG GCC TAC ATA CGG3’. FL, loxP-flanked allele; Δ932, deleted allele. Western blot analysis for wild type and Δ932 mutant CYLD expression in intestinal organoids **(d)** and epidermal keratinocytes **(f)** prepared from CYLD$\Delta932^{FL}$ and CYLD$\Delta932^{IEC}$ or CYLD$\Delta932^{epi}$ mice respectively. Note the slightly reduced MW of the truncated CYLD mutant (approximately 2,2 KDa).

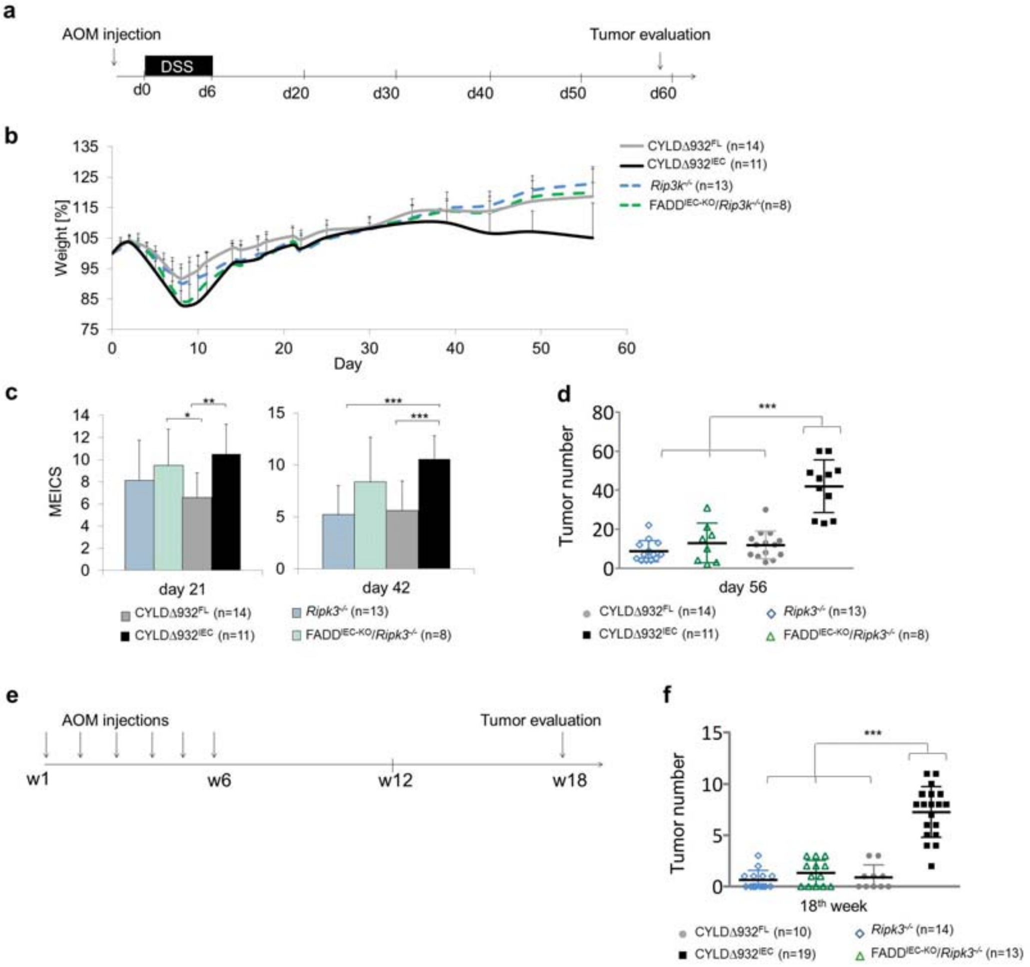

Supplementary Figure 2. The tumour suppressor function of CYLD is independent of RIPK3 and FADD/caspase-8 mediated cell death pathways

(a) CYLDΔ932^{IEC}, CYLDΔ932^{FL}, *Ripk3*$^{-/-}$, and FADD^{IEC-KO}/*Ripk3*$^{-/-}$ male mice were injected with 7,5 mg/kg AOM and 4 days later were given 2% DSS in the drinking water for 5 days. **(b)** Graph showing body weight changes throughout the treatment. **(c)** Quantification of murine endoscopic index of colitis severity (MEICS) on the indicated days. Data shown as mean ± SD. Statistical significance was determined with Student's *t*-test; *p≤0.05, **p≤0.005, ***p≤0.0005. **(d)** Graph showing colon tumour numbers on day 56. Pooled data from two independent experiments is shown. Similar results were obtained using female mice (data not shown). **(e)** CYLDΔ932^{IEC}, CYLDΔ932^{FL}, *Ripk3*$^{-/-}$, and FADD^{IEC-KO}/*Ripk3*$^{-/-}$ littermate mice were injected with 10 mg/kg AOM once a week for 6 weeks. **(f)** Graph showing colon tumour numbers on week 18th. Pooled data from two independent experiments is shown. Data shown as mean ± SD. Statistical significance was determined with Student's *t*-test; *p≤0.05, **p≤0.005, ***p≤0.0005. The cancer experiments presented in this figure where performed 2 years apart from the cancer experiments presented in Figure 1, using a different batch of AOM and DSS and a slightly different protocol, which generated a stronger response. Notice that for all our experiments our conclusions are based on the comparison between the responses of wild type versus mutant mice within the same experiment. All experiments were performed with littermates that were co-housed throughout the experiment.

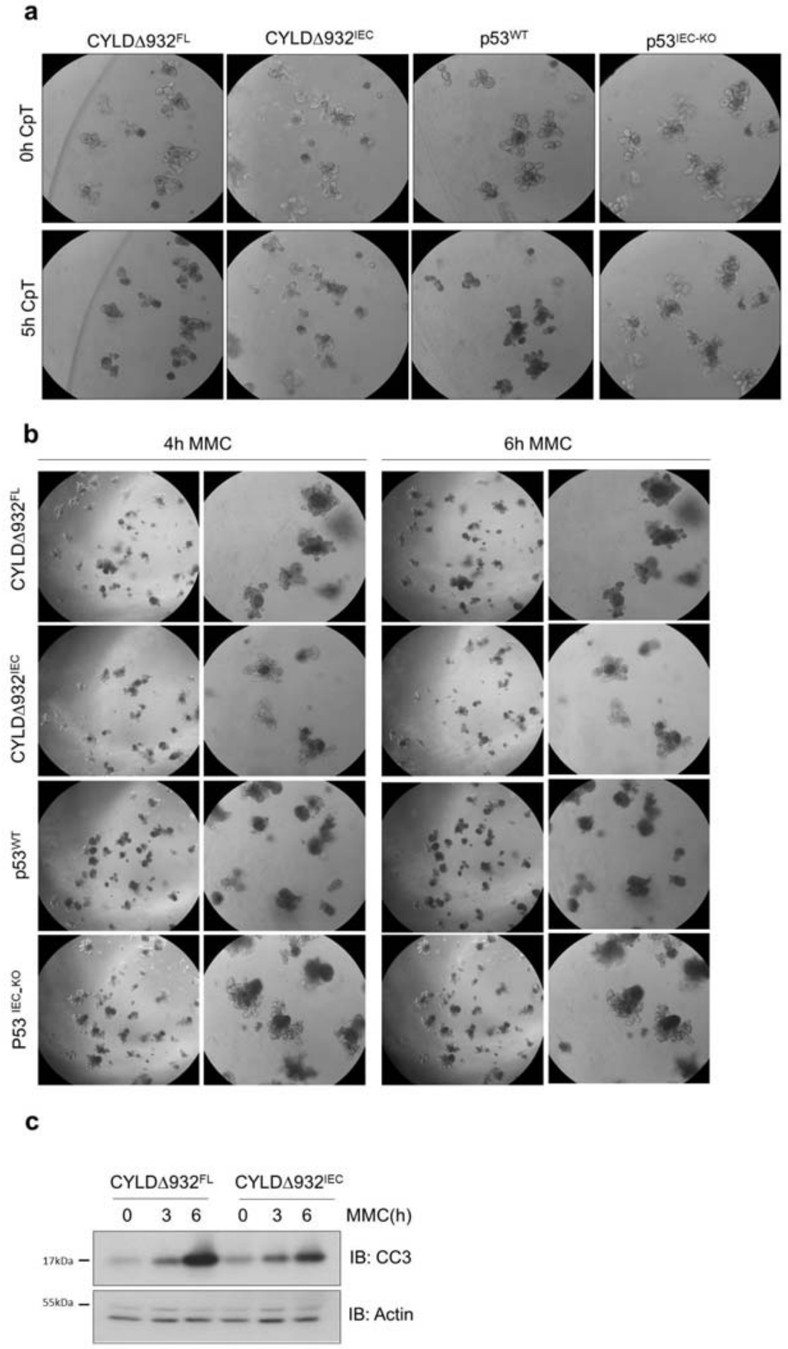

Supplementary Figure 3. CYLDΔ932 mutant intestinal organoids are protected from p53-dependent DNA damage induced cell death

Representative pictures of intestinal organoids prepared from CYLDΔ932^{FL}, CYLDΔ932^{IEC}, p53^{FL} and p53^{IEC-KO} mice treated with CpT **(a)** or MMC **(b)**. Pictures of the same organoids before and after treatment are shown at 40X and 100X magnification. **(c)** Immunoblot analysis for cleaved caspase-3 (CC3) in intestinal organoids prepared from CYLDΔ932^{FL}and CYLDΔ932^{IEC} mice treated with MMC for 3 and 6h.

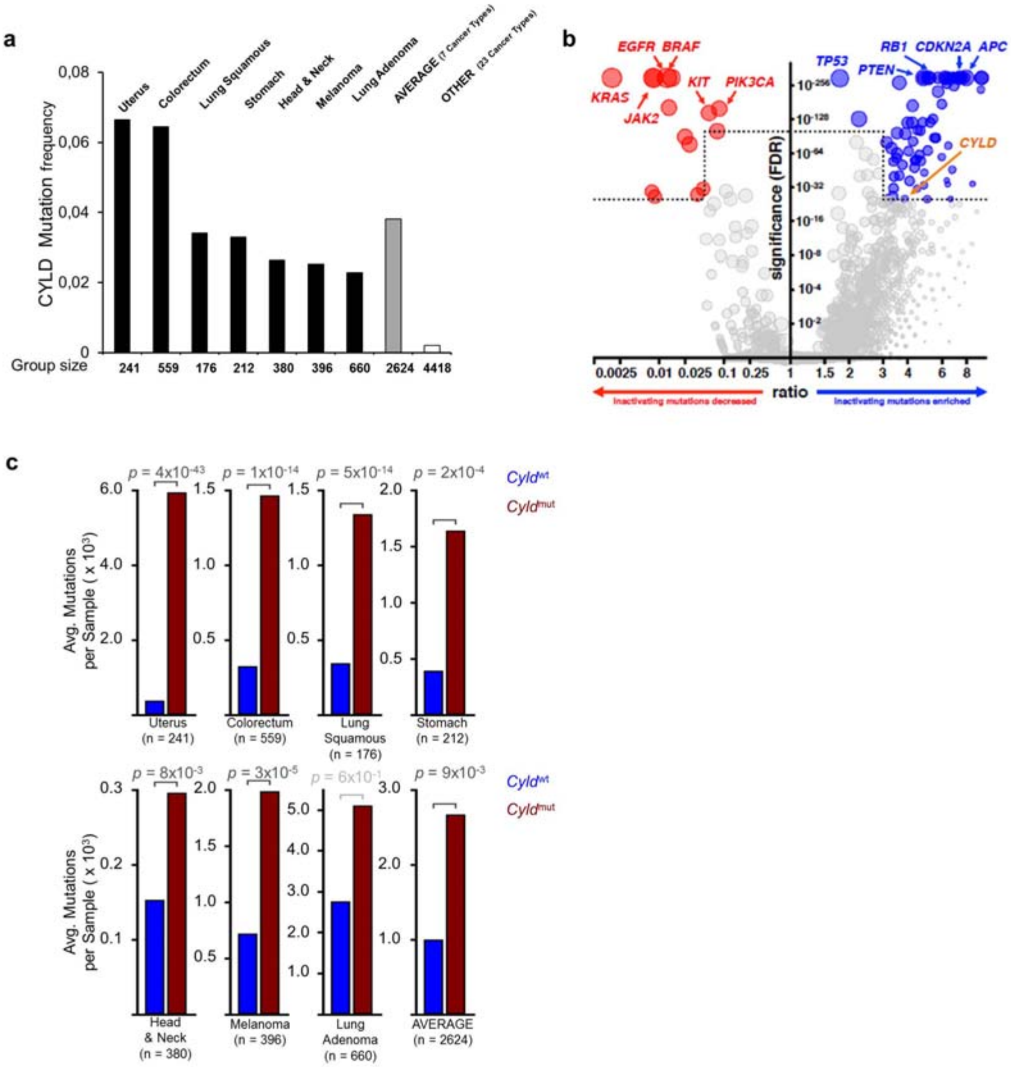

Supplementary Figure 4. Analysis of *Cyld* mutations in human cancer

(a) Frequency of somatic *Cyld* mutations across 7042 tumour samples from 30 distinct primary sites. Cancer types, for which *Cyld* mutations were recurrently detected, are depicted in black. Group size of each primary site is shown. **(b)** Volcano plot representation of a systematic analysis of mutation spectra in 27,836 genes (COSMIC database). Fraction of inactivating mutations was determined for each gene and tested for statistical significance assuming uniform distribution. The scatter plot displays for each gene (circles) the ratio of inactivating mutations normalized to average (x-axis) against the significance (Benjamini-Hochberg corrected false-discovery rate). Circle sizes encode overall mutation frequency. **(c)** Graphs showing the association between *Cyld* mutations and increase of the total number of mutations per sample. For each primary site, in which *Cyld* mutations were recurrently detected, the average number of mutations in *Cyld* mutant (red) vs. *Cyld* wt (blue) samples were compared by t-testing; p-values are shown.

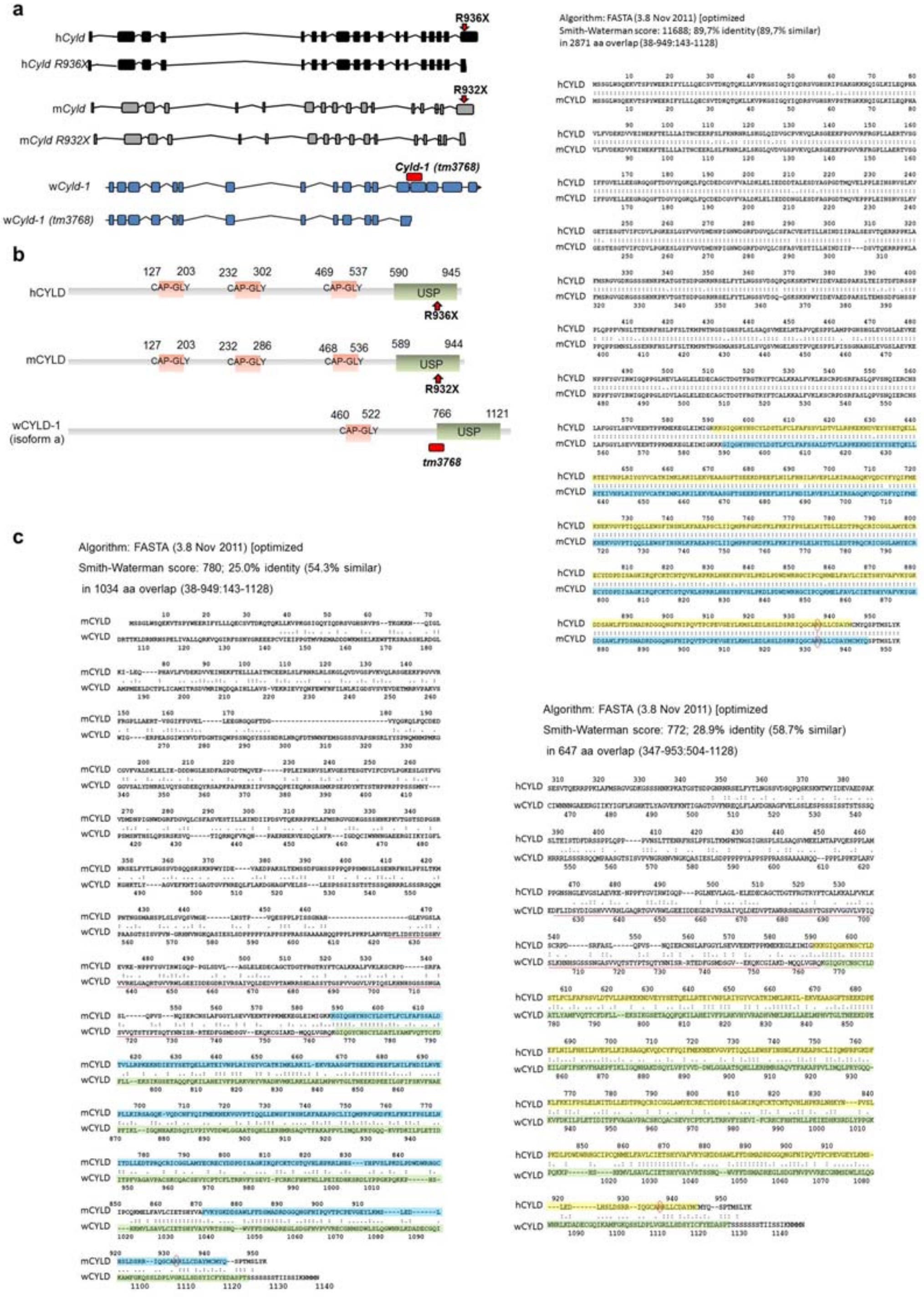

Supplementary Figure 5. Evolutionary conservation of CYLD

Schematic representation of the human (h), murine (m) and worm (w) wild type *Cyld* and the h*Cyld*R936X, m*Cyld*R932X and w*Cyld*TM3768 mutants genes **(a)** and proteins **(b)**. Exons and domains are shown. The position of mutations is indicated in red. **(c)** Alignment of the human, murine and worm protein using the algorithm FASTA (28 Nov 2001) optimized. Smith Waterman score and % of identity are shown. USP domains and mutations in each protein are highlighted. hCYLD USP domain: 590-945aa; mCYLD USP domain: 589-944aa; wCYLD USP domain: 766-1121aa. hCYLΔ936 mutation: aa substitution R936X; mCYLDΔ932 mutation: aa substitution R932X; wCYLDTM3768 mutation: deletion 623-764aa. aa: amino acid.

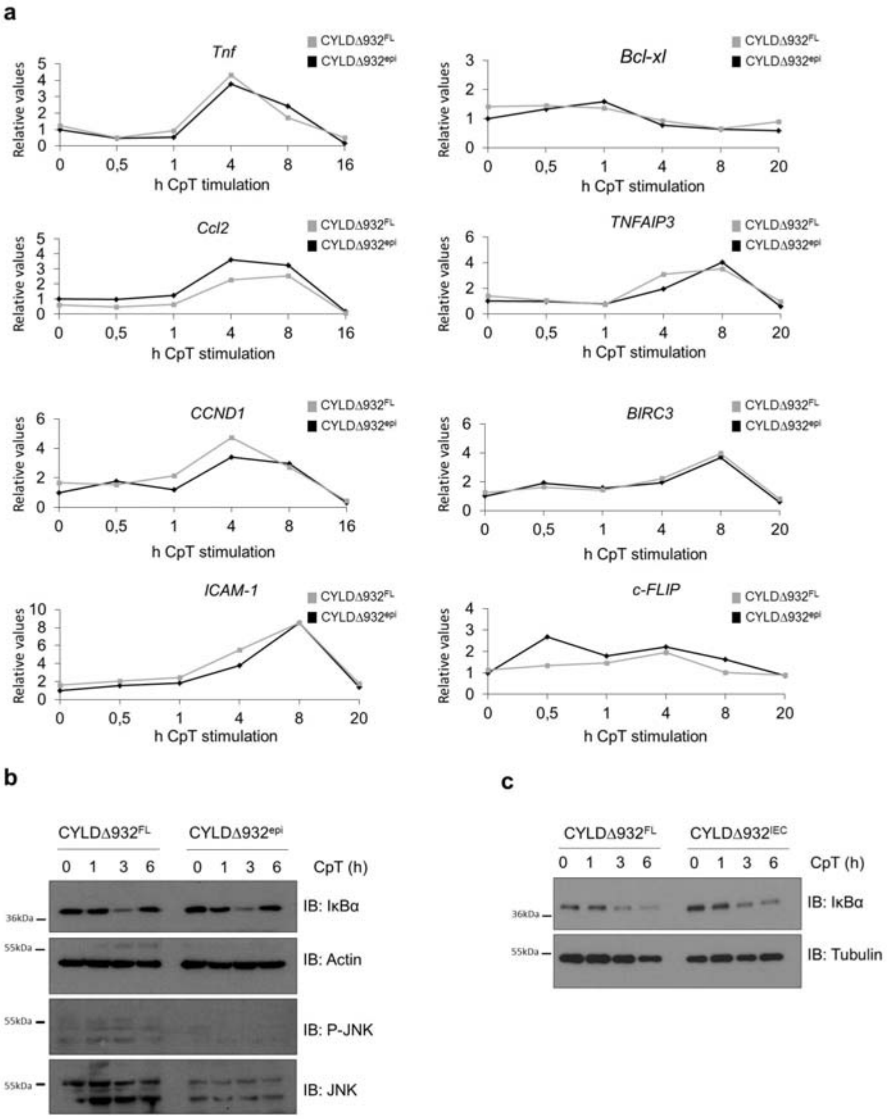

Supplementary Figure 6. DNA damage-induced NF-κB dependent gene transcription is not altered in CYLDΔ932 mutant primary cells

(a) qRT-PCR analysis of NF-κB target genes expression in primary epidermal keratinocytes from CYLDΔ932^{FL} and CYLDΔ932^{epi} mice treated with CpT for the indicated time points. Mean of technical replicates is shown. Immunoblot analysis for the indicated proteins in CpT-treated primary keratinocytes **(b)** and primary intestinal organoids **(c)** isolated from CYLDΔ932^{FL} and CYLDΔ932^{epi} or CYLDΔ932^{IEC} respectively.

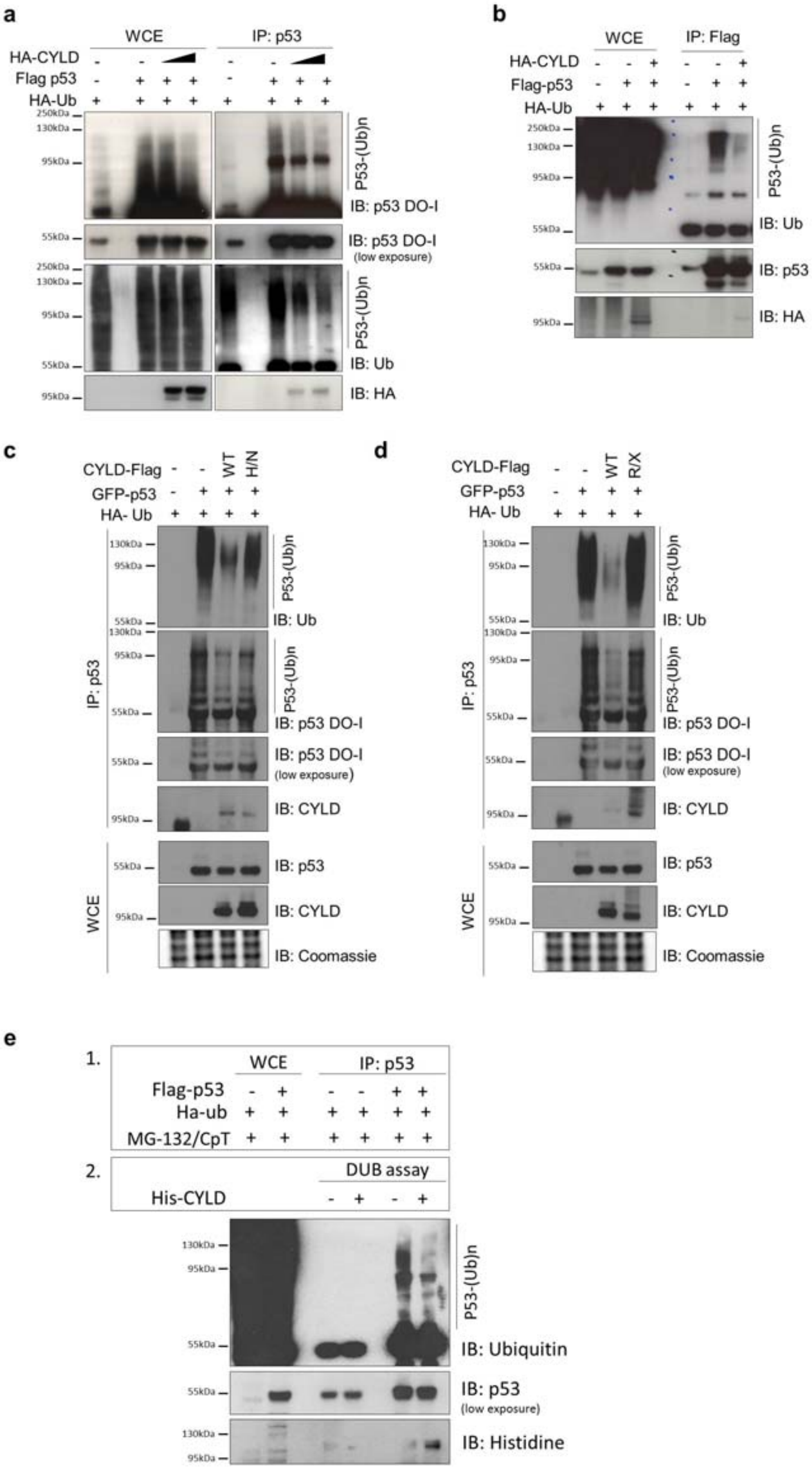

Supplementary Figure 7. CYLD binds and deubiquitinates p53

HEK-293T cells transfected with the indicated constructs were treated with MG132 and CpT for 7h and 3h respectively before harvested. p53 was immunoprecipitated with specific p53 antibodies **(a, c, d)** or Flag-M2 magnetic beads **(b)** followed by immunoblot with monoclonal anti-p53(DO-1), ubiquitin (Ub), CYLD and anti-HA antibodies. p53 and CYLD proteins expression levels in the wholes cell extracts (WCE) and levels of immunoprecipitated p53 are

shown in all panels. Coomassie Blue stainings were used as loading controls. **(e)** p53 was immunoprecipitated from HEK-293T cells transfected with the indicated constructs treated with MG-132/CpT and was subjected to an *in vitro* de-ubiquitination assay using 2μg of recombinant human Histidine-CYLD. Ubiquitinated levels of p53 after the assay are shown by immunoblot with anti-ubiquitin antibodies. Interaction of the recombinant His-CYLD with the immunoprecipitated p53 is shown by anti-Histidine immunoblot. Expression levels of p53 are shown in both the immunoprecipitates and the WCE. Coomassie staining or immunoblot for actin or tubulin served as loading controls.

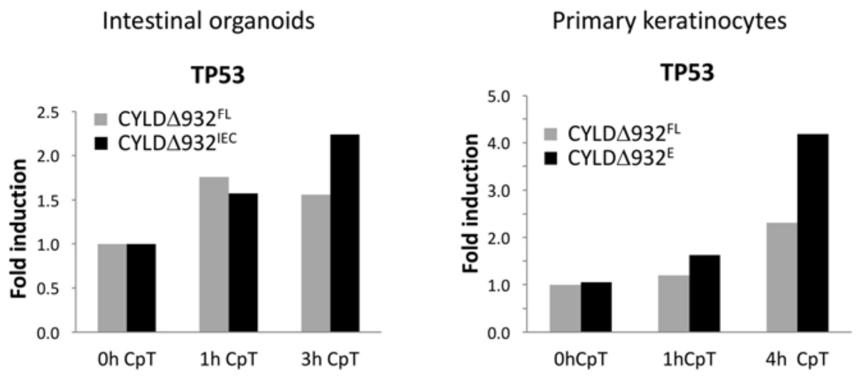

Supplementary Figure 8. Loss of CYLD catalytic activity does not inhibit TP53 mRNA expression.

qRT-PCR analysis of TP53 gene expression in intestinal organoids (left panel) or primary epidermal keratinocytes (right panel) from CYLDΔ932^{FL} and CYLDΔ932 mice treated with CpT for the indicated time points.

Figure 3B

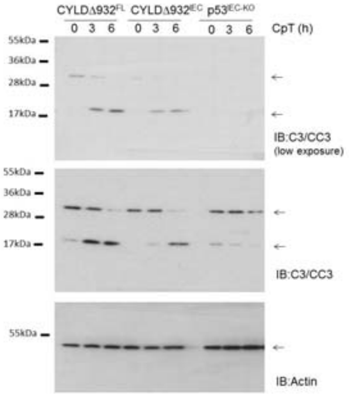

Figure 3E

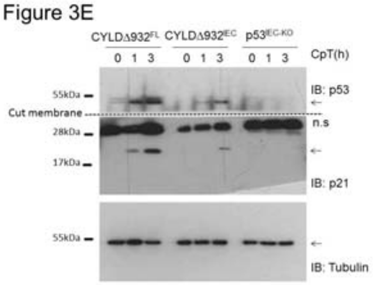

Figure 3G

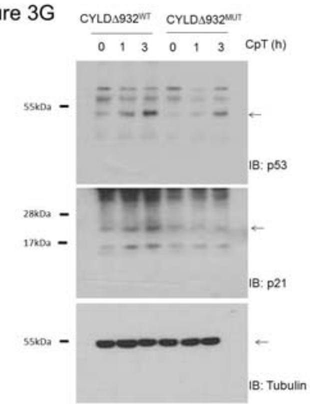

Figure 4A

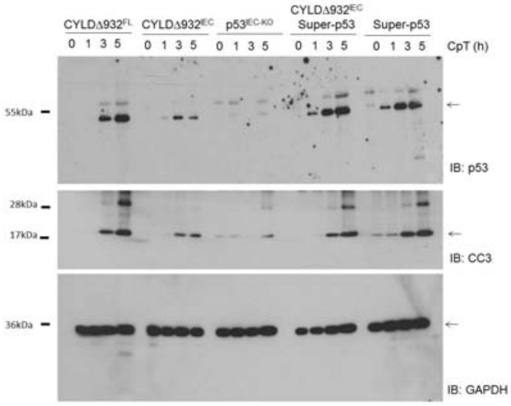

Figure 6A

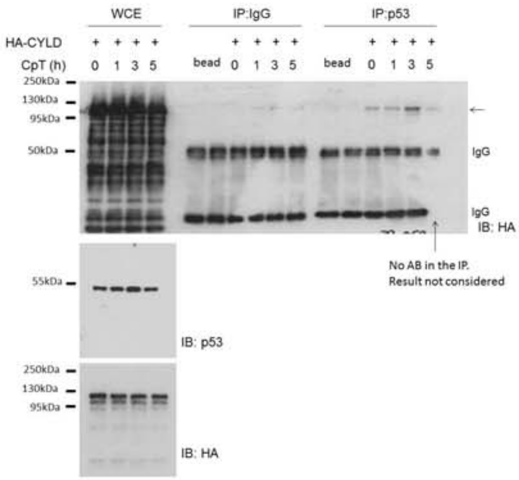

Figure 6B

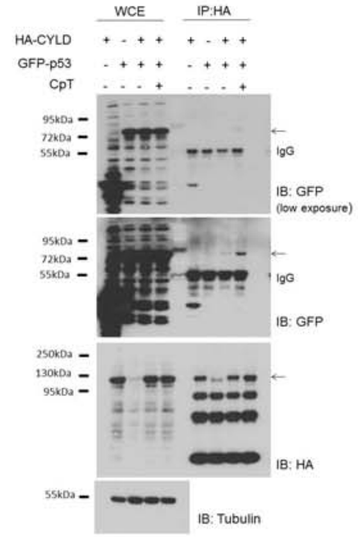

Figure 6C

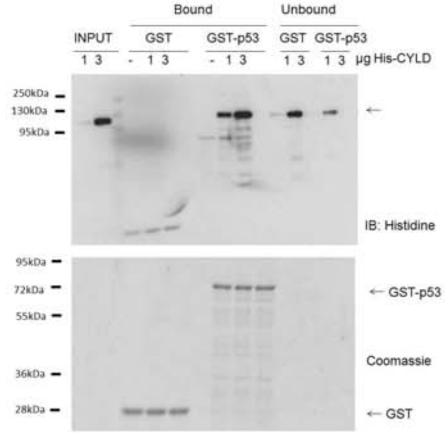

Figure 6D

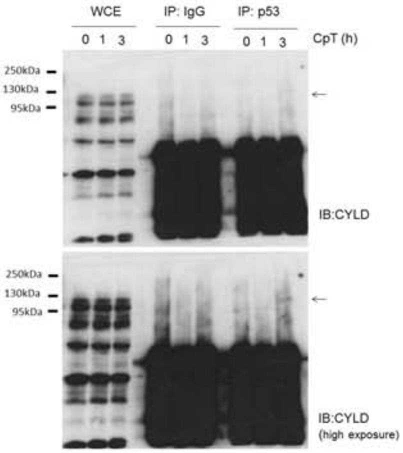

Figure 6E

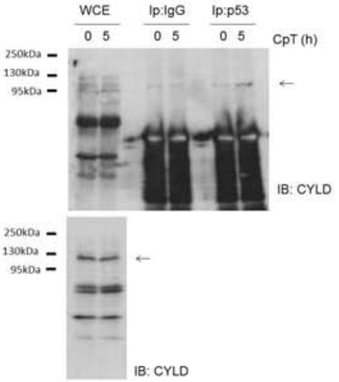

Figure 7A

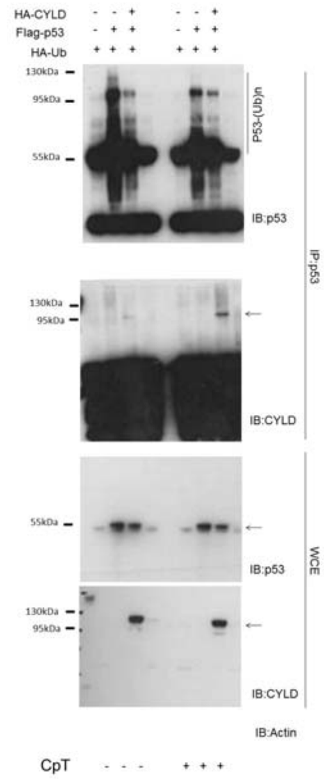

Figure 7B

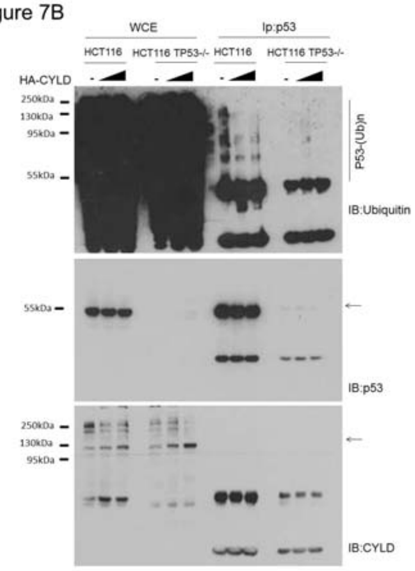

Figure 7C

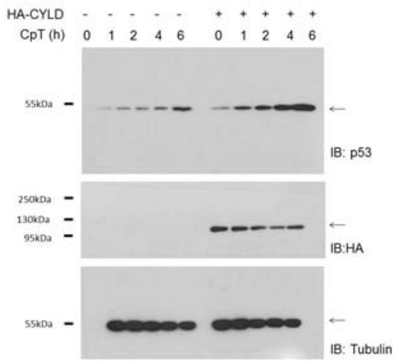

Figure 7D

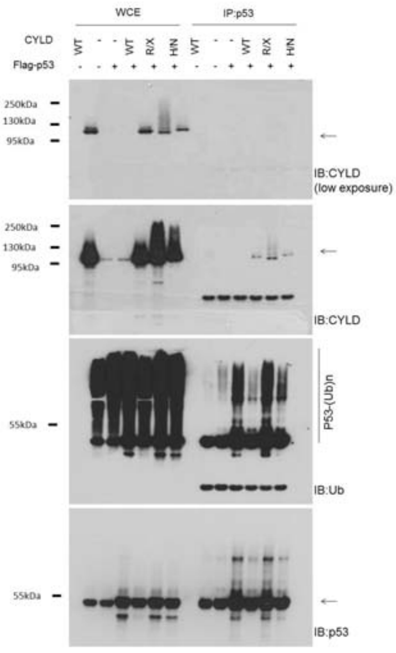

Figure 7E

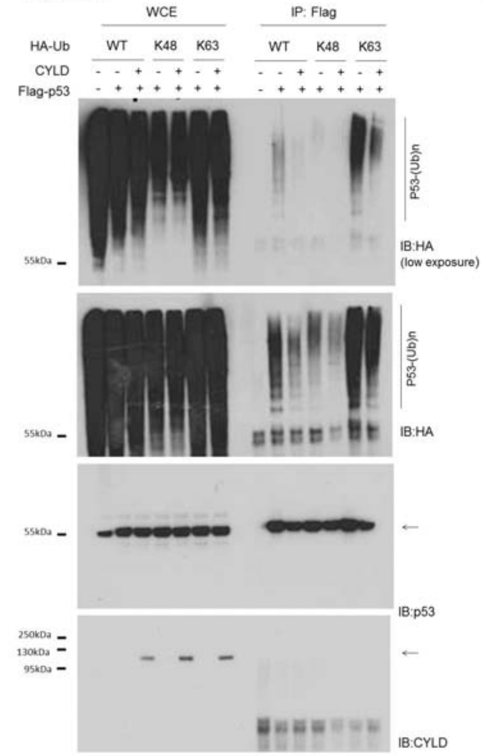

Figure 7F

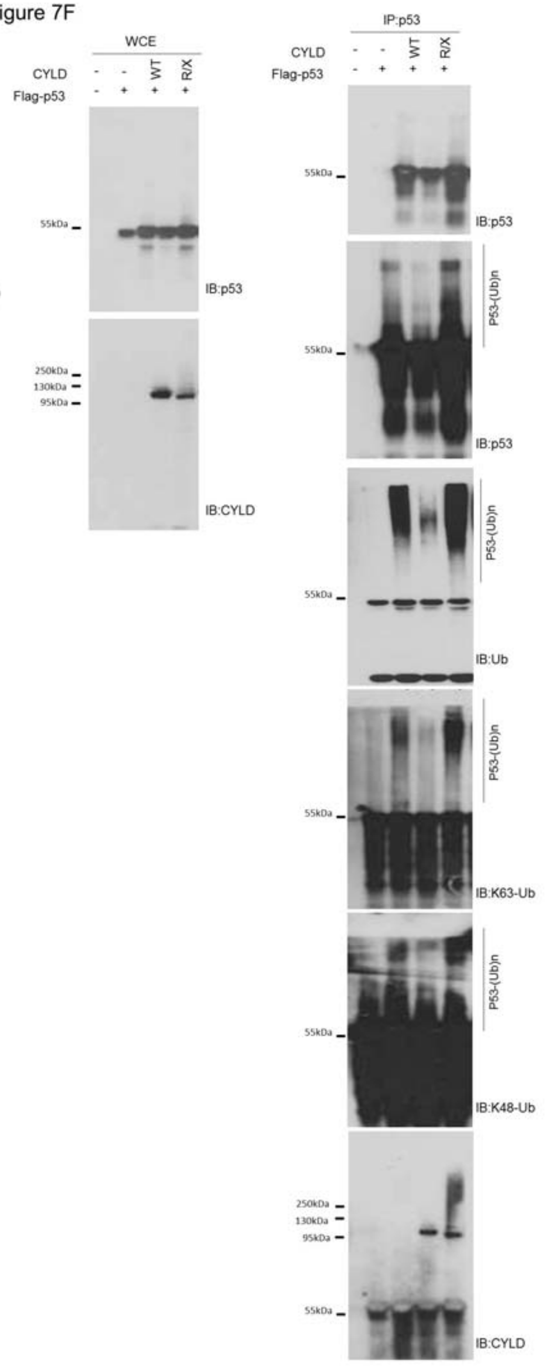

Figure 7G

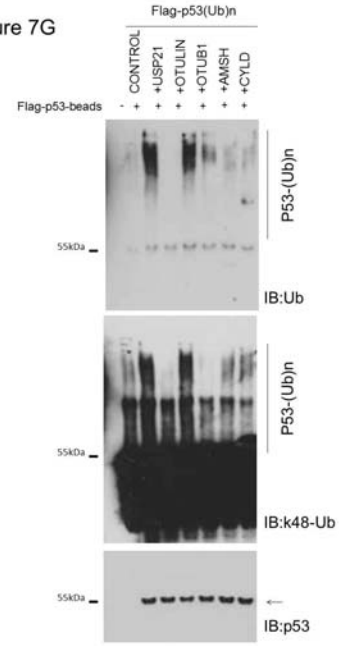

Supplementary Figure 9. Uncropped scans of western blots included in main figures.
